# Supplementary material for: Indecision and recency-weighted evidence integration in non-clinical and clinical settings
Source: Nat Hum Behav. 2026 Jan 21;10(4):727–40. doi: 10.1038/s41562-025-02385-1 (PMC13121037; doi:10.1038/s41562-025-02385-1)
Supplement: Supplementary file 2 — Reporting Summary [file 41562_2025_2385_MOESM2_ESM.pdf]

nature portfolio

Corresponding author(s): magdalena_del_rio@brown.edu & tobias.hauser@uni-tuebingen.de

Last updated by author(s): Oct 12, 2025

Reporting Summary

Nature Portfolio wishes to improve the reproducibility of the work that we publish. This form provides structure for consistency and transparency in reporting. For further information on Nature Portfolio policies, see our Editorial Policies and the Editorial Policy Checklist.

Statistics

For all statistical analyses, confirm that the following items are present in the figure legend, table legend, main text, or Methods section.

n/a	Confirmed	
☐	☒	The exact sample size (*n*) for each experimental group/condition, given as a discrete number and unit of measurement
☒	☐	A statement on whether measurements were taken from distinct samples or whether the same sample was measured repeatedly
☐	☒	The statistical test(s) used AND whether they are one- or two-sided<br>*Only common tests should be described solely by name; describe more complex techniques in the Methods section.*
☐	☒	A description of all covariates tested
☐	☒	A description of any assumptions or corrections, such as tests of normality and adjustment for multiple comparisons
☐	☒	A full description of the statistical parameters including central tendency (e.g. means) or other basic estimates (e.g. regression coefficient) AND variation (e.g. standard deviation) or associated estimates of uncertainty (e.g. confidence intervals)
☐	☒	For null hypothesis testing, the test statistic (e.g. *F*, *t*, *r*) with confidence intervals, effect sizes, degrees of freedom and *P* value noted<br>*Give P values as exact values whenever suitable.*
☒	☐	For Bayesian analysis, information on the choice of priors and Markov chain Monte Carlo settings
☒	☐	For hierarchical and complex designs, identification of the appropriate level for tests and full reporting of outcomes
☐	☒	Estimates of effect sizes (e.g. Cohen's *d*, Pearson's *r*), indicating how they were calculated

Our web collection on statistics for biologists contains articles on many of the points above.

Software and code

Policy information about availability of computer code

Data collection	Smartphone study data was collected using the Brain Explorer app and in-lab study data was collected using MATLAB.
Data analysis	Analysis scripts are publicly available at https://osf.io/fks97/

For manuscripts utilizing custom algorithms or software that are central to the research but not yet described in published literature, software must be made available to editors and reviewers. We strongly encourage code deposition in a community repository (e.g. GitHub). See the Nature Portfolio guidelines for submitting code & software for further information.

Data

Policy information about availability of data

All manuscripts must include a data availability statement. This statement should provide the following information, where applicable:

- Accession codes, unique identifiers, or web links for publicly available datasets
- A description of any restrictions on data availability
- For clinical datasets or third party data, please ensure that the statement adheres to our policy

Behavioural and summary neuroimaging data are publicly available at https://osf.io/fks97/.

Research involving human participants, their data, or biological material

Policy information about studies with human participants or human data. See also policy information about sex, gender (identity/presentation), and sexual orientation and race, ethnicity and racism.

Reporting on sex and gender	In the in-lab sample, sex was self-reported by participants. In the smartphone sample, gender was voluntarily self-reported by participants (i.e., data is not complete). No sex- or gender-based analyses were performed, as no hypotheses were sex- or gender-based.
Reporting on race, ethnicity, or other socially relevant groupings	No race, ethnicity, or other socially relevant groupings were used in either the smartphone population or the in-lab sample analysis.
Population characteristics	No additional population characteristics are included in the analysis for the smartphone population sample. Potentially relevant population characteristics for the in-lab sample include age, IQ, comorbidities, and medication status - these are not included in any analyses, but are compared across groups and reported in Supplementary Table 2.
Recruitment	The smartphone population sample was recruited through public engagement events and word of mouth to use the free app Brain Explorer (www.brainexplorer.net). There is self-selection bias in this sample, as participants are more likely to be engaged in science and/or OCD and mental health fields. The in-lab sample consisted of several groups with different recruitment strategies. OCD and GAD patients were recruited through NHS services, charities and advertisements. To ensure comparable backgrounds, controls were recruited in areas of similar socioeconomic status to the patients'. Participants from the high and low OC groups were recruited from a population-based sample of young people (U-CHANGE study; www.nspn.org.uk) based on their OCD questionnaire score. There is self-selection bias in this sample insofar as participation requires patients to be able and willing to be included, limiting the severity and/or type of symptom profiles represented in the sample. We do not consider any of these biases likely to significantly impact the results.
Ethics oversight	University College London (UCL) and NHS research ethics committees

Note that full information on the approval of the study protocol must also be provided in the manuscript.

Field-specific reporting

Please select the one below that is the best fit for your research. If you are not sure, read the appropriate sections before making your selection.

☐ Life sciences ☒ Behavioural & social sciences ☐ Ecological, evolutionary & environmental sciences

For a reference copy of the document with all sections, see nature.com/documents/nr-reporting-summary-flat.pdf

Behavioural & social sciences study design

All studies must disclose on these points even when the disclosure is negative.

Study description	Data are quantitative - comprising self-report, behavioural and neurophysiological (MEG) data.
Research sample	The smartphone population sample is a sample self-selected from the general population, in order to include variability along demographic, cognitive and mental health measures at scale.<br>The in-lab sample consisted of clinically diagnosed OCD and GAD patients, healthy controls and undiagnosed individuals with low and high levels of OCD-like symptoms (as assessed by data available from the U-CHANGE study, see above). The rationale was to include variability along obsessive-compulsive as well as anxiety-related dimensions, relevant to information gathering.
Sampling strategy	The smartphone population sample is a convenience sample, self-selected from the general population. The sample size was determined by the population's participation - post hoc sensitivity calculations using G*Power show that the sample of 5,237 is able to detect correlation effects of size 0.04 with 80% power and 0.05 error probability, which is the smallest reported significant effect size. The in-lab sample was selectively sampled. Sample sizes in this case were determined by prior studies as well as pragmatic matters (recruitment and financial limitations).
Data collection	The smartphone study was conducted remotely by participants on smartphone devices, such that researchers were not present and no additional details are available.<br>The in-lab study was conducted with the researcher present. During the interviews, participants and researchers used pen and paper and computers to record responses and comments. During the task, participants had electrophysiological neural signals recorded using a MEG scanner and recorded their responses using a MEG-compatible device. Researchers were not blind to the study hypotheses and experimental conditions were constant across participants during data collection.
Timing	The smartphone sample data was collected between November 2020 and August 2023. The in-lab sample data was collected between April 2015 and August 2018.
Data exclusions	In the smartphone population analysis, 1,927 participants were excluded because of missing questionnaire data. An additional 1,506 participants were excluded based on performance metrics determined by the analysis requirements. Exclusion criteria were not pre-established.

	In the in-lab sample analysis, two participants were excluded because they did not meet the clinical criteria for GAD, one participant was excluded because of comorbid OCD and GAD, one was excluded due to fully missing questionnaire data (pre-established exclusion criteria). One participant was excluded due to performance-related metrics, and five participants were excluded due to technical difficulties and/or poor MEG data quality (not pre-established exclusion criteria).
Non-participation	There is no record of participants dropping out or declining participation for the smartphone study. No participants dropped out of the in-lab study. Participants who declined participation in the in-lab study were not recorded.
Randomization	Participants were not allocated to experimental groups.

Reporting for specific materials, systems and methods

We require information from authors about some types of materials, experimental systems and methods used in many studies. Here, indicate whether each material, system or method listed is relevant to your study. If you are not sure if a list item applies to your research, read the appropriate section before selecting a response.

Materials & experimental systems

n/a	Involved in the study
☒	☐ Antibodies
☒	☐ Eukaryotic cell lines
☒	☐ Palaeontology and archaeology
☒	☐ Animals and other organisms
☒	☐ Clinical data
☒	☐ Dual use research of concern
☒	☐ Plants

Methods

n/a	Involved in the study
☒	☐ ChIP-seq
☒	☐ Flow cytometry
☒	☐ MRI-based neuroimaging

Plants

Seed stocks	*Report on the source of all seed stocks or other plant material used. If applicable, state the seed stock centre and catalogue number. If plant specimens were collected from the field, describe the collection location, date and sampling procedures.*
Novel plant genotypes	*Describe the methods by which all novel plant genotypes were produced. This includes those generated by transgenic approaches, gene editing, chemical/radiation-based mutagenesis and hybridization. For transgenic lines, describe the transformation method, the number of independent lines analyzed and the generation upon which experiments were performed. For gene-edited lines, describe the editor used, the endogenous sequence targeted for editing, the targeting guide RNA sequence (if applicable) and how the editor was applied.*
Authentication	*Describe any authentication procedures for each seed stock used or novel genotype generated. Describe any experiments used to assess the effect of a mutation and, where applicable, how potential secondary effects (e.g. second site T-DNA insertions, mosiacism, off-target gene editing) were examined.*
